# The first ITS1 profiling of honey samples from the Southeast Asian region Lombok, Bali and Banggi Island

**DOI:** 10.1038/s41598-024-64838-3

**Published:** 2024-06-19

**Authors:** Saeed Ullah, Fahrul Huyop, Roswanira Ab. Wahab, Nurul Huda, Habeebat Adekilekun Oyewusi, I. Gede Arya Sujana, Satrijo Saloko, Anak Agung Sagung Putri Risa Andriani, Mohd Azrul Naim Mohamad, Azzmer Azzar Abdul Hamid, Mohd Hamzah Mohd Nasir, Nyoman Semadi Antara, Ida Bagus Wayan Gunam

**Affiliations:** 1 Department of Biosciences, Faculty of Science, Universiti Teknologi Malaysia, 81310 Johor Bahru, Malaysia; 2 Department of Chemistry, Faculty of Science, Universiti Teknologi Malaysia, 81310 Johor Bahru, Malaysia; 3 Faculty of Sustainable Agriculture, Universiti Malaysia Sabah, 90509 Sandakan, Sabah Malaysia; 4 Faculty of Food Technology and Agro Industry, University of Mataram, Mataram, Nusa Tenggara Barat 83126 Indonesia; 5 Faculty of Agriculture, Warmadewa University, Denpasar, Indonesia; 6 Research Unit for Bioinformatics and Computational Biology (RUBIC), Kulliyyah of Science, International Islamic University Malaysia, Bandar Indera Mahkota, 25200 Kuantan, Pahang Malaysia; 7 Biochemistry Unit, Department of Science Technology, The Federal Polytechnic, P. M. B 5351, Ado Ekiti, Ekiti State Nigeria; 8 Present Address: Bioindustry Laboratory, Department of Agro-Industrial Technology, Udayana University, Denpasar, Indonesia

**Keywords:** ITS1, ASVs, Bali, Lombok, Banggi, Illumina sequencing, Computational biology and bioinformatics, Molecular biology

## Abstract

Southern Asian flowers offer honeybees a diversity of nectar. Based on its geographical origin, honey quality varies. Traditional methods are less authentic than DNA-based identification. The origin of honey is determined by pollen, polyphenolic, and macro-microorganisms. In this study, amplicon sequencing targets macro-microorganisms in eDNA using the ITS1 region to explore honey’s geographical location and authentication. The variety of honey samples was investigated using ITS1 with Illumina sequencing. For all four honey samples, raw sequence reads showed 979,380 raw ITS1 amplicon reads and 375 ASVs up to the phylum level. The highest total number of 202 ASVs up to phylum level identified Bali honey with 211,189 reads, followed by Banggi honey with 309,207 a total number of 111 ASVs, and Lombok represents only 63 ASVs up to phylum level with several read 458,984. Based on Shannon and Chao1, honey samples from Bali (B2) and (B3) exhibited higher diversity than honey from Lombok (B1) and green honey from Sabah (B4), while the Simpson index showed that Banggi honey (B4) had higher diversity. Honey samples had significant variance in mycobiome taxonomic composition and abundance. *Zygosaccharomyces* and *Aspergillus* were the main genera found in Lombok honey, with percentages of 68.81% and 29.76% respectively. Bali honey samples (B2 and B3) were identified as having a significant amount of the genus *Aureobasidium*, accounting for 40.81% and 25% of the readings, respectively. The microbiome composition of Banggi honey (B4) showed a high presence of *Zygosaccharomyces* 45.17% and *Aureobasidium* 35.24%. The ITS1 analysis effectively distinguishes between honey samples of different origins and its potential as a discriminatory tool for honey origin and authentication purposes.

## Introduction

The floral source determines the honey’s color, flavor, and aroma, by the distinctions between light and mild clover honey and dark, robust buckwheat honey. Mineral content in nectar influences honey’s physical properties, including electrical conductivity. Furthermore, plant-derived secondary metabolites in nectar contribute to honey’s antioxidant and antibacterial qualities, imparting unique floral nuances^1–3^. Nectar, the primary resource for honey production, exhibits a complex composition crucial for sustaining bee colonies.

Comprising sugars in the range of 20–80%, with sucrose, fructose, and glucose serves as a concentrated energy source for bees. Additionally, nectar contains 70–80% water, requiring enzymatic processing by honeybees to reduce it for prolonged storage. Trace elements such as amino acids, proteins, vitamins, minerals, and secondary metabolites contribute to honey’s distinctive flavor, color, and antibacterial attributes. The search for honey of the highest caliber, free of any human contamination, and abundant in natural constituents is of utmost importance^4^. Honey, mainly for its high osmolarity, low water activity, natural acidity, and low protein content, does not favor the growth of microorganisms^5^.

The microbes acquired in honey are likely derived from many sources, including pollen, soil, dust, water, Meliponini’s digestive tract, air, and nectar, which are challenging to control. Furthermore, inadequate attention to hygiene during honey processing, handling, and storage can result in post-harvest microbial contamination^6^. Fungi are a prominent Kingdom within the Eukaryotic sphere of life. Certain species possess significant economic value as they produce antibiotics, aid in the fermentation of food products like beer and bread, and facilitate the breakdown of cellulose. The number of fungal species is expected to be in the millions, yet only a limited few have been thoroughly described^7^. Fungi that are frequently present in plants and soil can be transferred to beehives via the process of pollination. The plant-associated microbes can be found in honey and other bee products such as bee bread, which is a fermented mixture of pollen and nectar used as food for bees. Some of these microbes have a positive impact on the bee colonies^8^.

The mycobiome refers to the fungal part of the microbiome. The term was initially employed specifically about the human mycobiome^7^. Yeast was the dominant fungi in honey identified using culture and molecular-based methods, such as *Zygosaccharomyces, Debaryomyces*, and *Candida*, have been previously identified in honey^9^. The ubiquitous *Aspergillus*, functioning as an opportunistic pathogen, poses a threat to honeybee larvae and triggers stonebrood disease. *Ascosphaera apis*, the causative agent of chalkbrood disease, and spore-forming fungi *Nosema apis* and *Nosema ceranae* are contributors to nosema disease^10,11^. One of the most prevalent types of fungi identified in honey is *Cladosporium*, a filamentous fungus that is ubiquitous and, according to some assumptions, could even live in concord with bees, spread from plant to bee, and then settle into bee products^8^. Honey is often contaminated with filamentous fungi, such as *Cladosporium, Alternaria*, and *Aspergillus*^9^.

The chemical features of honey, such as its low water activity, low pH, and presence of hydrogen peroxide, prevent the growth of microorganisms. As a result, latent forms of microbes, such as bacterial spores and yeasts, are commonly noticed in honey. The particles will originate from the flowers that the bees visit, as well as from the immediate environment of the hive (such as the soil and water), and from the hive and the bees themselves. Therefore, they can potentially offer additional evidence regarding the sources of honey^3^.

Few studies have investigated fungal species on bees, however functional studies show that yeasts impact bee growth, pathogen interactions, and foraging. Most fungi are either facultative bees associated with uncertain or environmentally contextual effects; the rare ones are obligately beneficial symbionts^12^. Honey contains eDNA from both microbes and bees, offering valuable insights into the hive microbiome and the honeybee hologenome. Consequently, eDNA analysis sheds light on bee pathospheres and overall colony health^13–16^. To refine the source attribution, incorporating data from diverse taxonomic groups detected in honey alongside plant-derived information allows for the application of DNA-based techniques to comprehensively identify the honey’s microbial constituents^2,3^.

Analyzing the honey microbiome may illuminate beehive dynamics, bolster honey production, and combat honeybee diseases. However, prior studies predominantly employ culture-based methodologies, which may entail culture-driven biases^17^. Culture-independent techniques, exemplified by next-generation sequencing, have begun to explore honeybee-associated microbial realms, unveiling insights into the microbiome of honeybee gastrointestinal tracts, pollen, and bee bread, although metagenomic analyses of honey remain limited^10,14,18–20^.

The DNA traces provide evidence of the micro macro-organisms that the bees have come into physical contact with, whether it was intentional or unintentional. Many studies suggest that identification of plant, fungal and bacterial taxonomic groups play a role as in identification of honey origin and separating honey samples of different origin^3^. Thus, the purpose of this study to demonstrate the prospect of DNA-based approaches in determining a fungal taxonomic group in newly selected area. To thoroughly examine the presence of micro and macro-organisms (specifically the fungal community) in honey and to provide a comprehensive understanding of honey quality, authentication from various locations in Lombok, Bali and Banggi Island. A culture-independent method ITS1 was utilized to obtain a detailed description of the fungal microbiota present in the honey samples. The microbial fingerprints of honey, particularly its fungal diversity, hold immense potential for revealing its origin and floral sources.

This study aims to examine the ITS1 diversity (alpha and beta) of honey samples from distinct regions—Lombok, Bali and Banggi. Ultimately, this investigation aspires to translate its findings into practical recommendations for safeguarding the regional microbial diversity paving the way for sustainable and authentic honey production practices.

## Results

### Amplicon data sequencing summary

A total of 979,380 raw reads were obtained from 12 samples, with the average raw reads and filtered reads detailed in (Table 1). Variations in G + C content (%) were observed among the samples. Bali honey (B2 and B3) exhibited the highest G + C content, suggesting a relatively higher proportion of guanine and cytosine nucleotides in its genetic makeup. In contrast, Banggi honey (B4) and Lombok (B1) showed comparatively lower G + C content, indicating distinct genetic characteristics (Table 1). These findings highlight the diversity and variability present in the sequence data obtained from different honey samples, providing insights into their genetic composition and potential differences between them.

**Table 1:** Information of raw Sequence read for honey samples by NCBI Annotation Pipeline.

Attribute	Lombok (B1)	Bali (B2)	Bali (B3)	Banggi (B4)
N_50_ (bp)	69,446	42,696	73,496	51,039
Size (MB)	3.5	2.5	3.5	2.5
G + C content (%)	46	55	48.30	45
Sequence read archive	SAMN38113445	SRR26725285	SRX22424191	SRR26725282
Bio sample accession	SRR26725283	SAMN38113443	SAMN38113443	SAMN38113446
Bio project accession	PRJNA1036045	PRJNA1036045	PRJNA1036045	PRJNA1036045
Number of samples	4	2	2	4
Number of raw reads	458,984	90,311	120,878	309,207
Number of filters reads	411,394	56,374	70,806	239,487
Average filtered raw reads	102,848 ± 40,577	28,187 ± 12,105	35,403 ± 2,729	59,871 ± 27,206
Number of ASVs	63	100	102	111
Average ASVs	35 ± 6	59 ± 16	60 ± 25	52 ± 16

Table 1 also provides a summary of the sequencing data obtained from honey samples collected from four distinct locations. The table outlines the number of samples analyzed from each location, along with the corresponding counts of raw reads and filtered reads after eliminating unidentified sequences of ITS1 amplicon reads. The data reveals notable differences in the quantity of reads obtained per sample across the locations. Lombok honey samples (B1) exhibited the highest average number of filtered sequencings reads per sample, followed by Banggi honey samples (B4).

In contrast, Bali honey samples (B2) displayed the lowest average number of filtered sequencings reads per sample, with a significantly lower value compared to the other locations. Moreover, the data presents the number of amplicon sequence variants (ASVs) identified for each type of honey sample. Despite similar values across locations, Banggi honey samples showed the highest average number of ASVs per sample. However, it is important to note the relatively high standard deviations for all locations, indicating variability in the bacterial and fungal communities present in honey samples from these locations.

### Alpha diversity (richness) analysis of honey samples

Table 2 provides a comprehensive overview of alpha diversity analysis conducted on honey samples collected from Lombok (B1), Bali (B2 and B3), and Banggi (B4). Alpha diversity, which denotes the variety of species within a single location, was assessed using multiple indices, including Chao1, Shannon, and Simpson. Chao1 Index specifies the Bali honey samples (B2) exhibited the highest estimated total number of species with a Chao1 value of 60.00 ± 31.11, followed by Bali (B3) (58.00 ± 19.79), Banggi (B4) (52.67 ± 16.36), and Lombok (B1) (35.62 ± 7.18). This suggests a potential gradient in species richness, with Bali (B2) harbouring the most diverse microbial community.

**Table 2:** Represent alpha diversity for honey samples based on Chao1, Shannon and Simpson index.

	Lombok (B1)	Bali (B2)	Bali (B3)	Banggi (B4)
Chao1	35.62 ± 7.18	60.00 ± 31.11	58.00 ± 19.79	52.67 ± 16.36
Shannon	1.59 ± 0.058	4.02 ± 1.88	4.63 ± 1.19	1.87 ± 1.33
Simpson	0.51 ± 0.038	0.78 ± 0.22	0.89 ± 0.092	0.39 ± 0.24

Shannon Index demonstrated Bali (B3) the highest evenness with a Shannon index value of 4.63 ± 1.19, followed by Bali (B2) (4.02 ± 1.88). B2 and B3 located in the same island. Lower values were observed in Banggi (B4) (1.87 ± 1.33) and Lombok (B1) (1.59 ± 0.058), indicating a less even distribution of species in these samples.

Simpson Index on Banggi honey (B4) displayed the highest diversity (0.39 ± 0.24), followed by Lombok (B1) (0.51 ± 0.038). Conversely, Bali honeys (B2 and B3) had lower values (0.78 ± 0.22 and 0.89 ± 0.092, respectively), indicating a higher dominance of specific microbial species in these samples. Combining the analysis of Chao1, Shannon, and Simpson indices, honey samples from Bali (B2) exhibit the highest overall alpha diversity in terms of species richness. However, Bali (B3) demonstrates a more balanced distribution of species within the community, as indicated by the Shannon index. Interestingly, Banggi honey (B4) displays the highest diversity based on the Simpson index, suggesting a lower dominance of specific microbial species. It is important to acknowledge the limitations of this analysis. Standard deviations associated with the diversity indices indicate variability within each location. Additionally, factors like sequencing depth and potential sampling biases might influence the observed diversity patterns.

### Beta diversity analysis of honey samples

**Figure 1:**
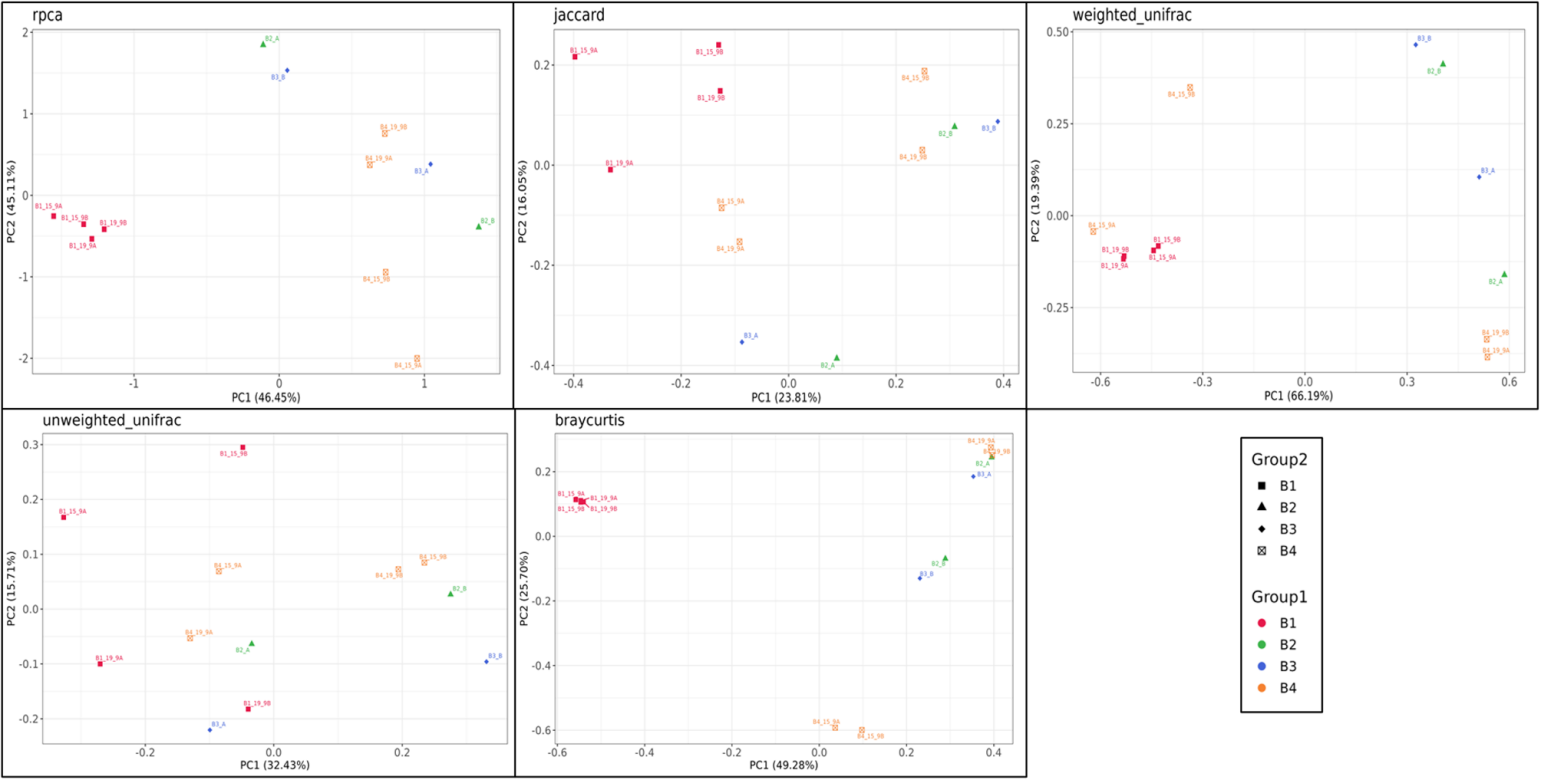
Beta diversity represented by the rpca, Bray Curtis, Jaccard, weightated_unifarc and unweighted_unifarc. Each plot point represents a single sample and was colored and shaped by group according to the legend. Samples that clustered close together are more likely to share a similar microbial composition.

Beta diversity of honey samples from different sources was calculated using robust Aitchison, Principal Coordinate Analysis (PCoA), Bray–Curtis, Jaccard, weighted Unifrac, and unweighted Unifrac methods (Fig. 1). Beta diversity quantifies the variation in species composition among locations or habitats within a broader region. It complements alpha diversity, which refers to the variety within a single community.

In our investigation, we noticed that honey samples forming tight clusters in the Principal Coordinate Analysis (PCoA) plot shared more similar mycobiome compositions, while those scattered further apart exhibited greater dissimilarities. Each location exhibited a distinct signature of genera and species, as evidenced by variations in Beta diversity metrics such as Bray–Curtis, Jaccard, weighted UniFrac, and unweighted UniFrac. These findings imply that environmental factors such as geography, flora, and fauna might influence the spatial diversity in honeybee foraging patterns and microbial composition. In the PCoA analysis, the proximity of points on the plot denotes similarities in fungal microbiota composition, while greater distances indicate more pronounced differences in fungal species types and quantities. The arrangement of individual samples on the plot reflects disparities in mycobiome composition, with the two principal components accounting for 91.56% of the variance, thereby representing the most significant dimensions of differentiation among the honey samples. Bali (B3) stands out prominently above Lombok (B1) and Bali (B2) due to its distinct fungal ecology, resulting in discernible plot segregation. Although the fungal community in Banggi (B4) closely resembles that of Bali (B3), its high-abundance taxa form a distinct cluster (Fig. 1).

The Bray–Curtis index, while primarily influenced by dominant taxa, is relatively less swayed by their dominance in honey samples. Approximately 74.98% of dissimilarities were elucidated by the two principal components analysis. In the plot, Lombok honey (B1) was positioned on the left side, while Banggi honey (B4) occupied the right side, indicating stark dissimilarities between these samples. Conversely, Bali (B3) and Banggi (B4) appeared relatively closer to each other in the middle of the plot, suggesting similarities in mycobiota and origin between these honey samples.

Regarding the Jaccard index, utilized for comparing the similarity between distinct groups, approximately 39.86% of similarities were explained by the first two principal components among all honey samples. A Jaccard index value close to 0 or negative suggests greater dissimilarities than similarities between samples from different groups, implying diverse fungal compositions among honey samples from different groups.

Weighted UniFrac is biased toward measuring abundant taxa in samples. Bali (B2) and Banggi (B4) honey samples exhibit greater diversity, with the highest value of 0.6 at PC2, followed by Bali (B3), and the lowest in Lombok (B1) honey. This suggests distinct fungal communities across all honey samples.

In contrast, the Unweighted UniFrac index is inclined towards rare taxa and is less sensitive to abundance variations. Based on this index, all honey samples appear more diverse and overlap with each other. The comprehensive analysis of beta diversity measurements through PCoA and various indices provides valuable insights into the unique microbial compositions of honey samples from different geographical regions. These differences likely arise from environmental factors influencing honeybee foraging behaviors and subsequent microbial populations in the honey.

### Mycobiome community in honey

**Figure 2:**
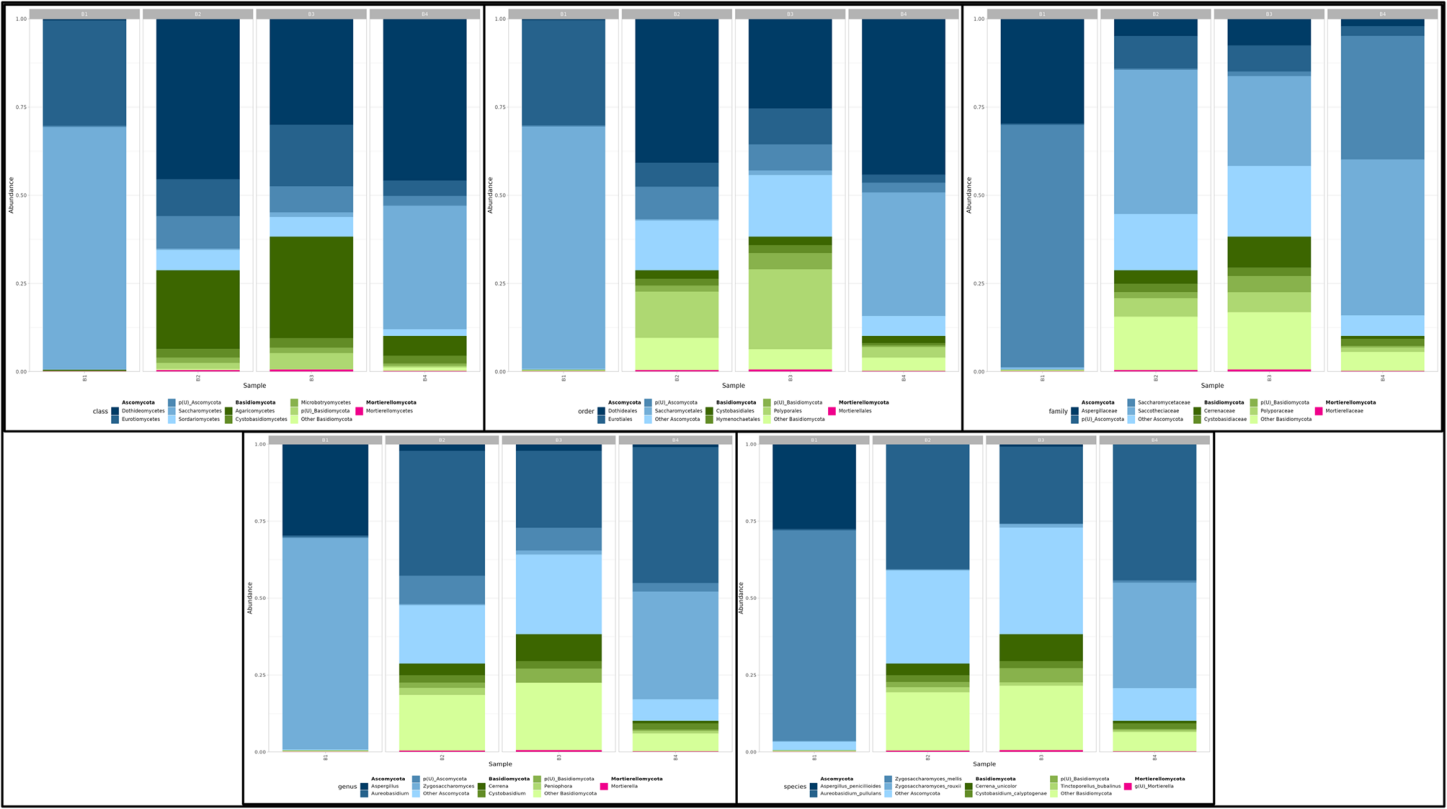
Honey fungal abundance plot at the Phyla/Class (Supplementary file S1), Order, Family, Genus/Species level. For every honey sample, ASVs were aggregated to the genus level. For this plot, we only used species with a relative abundance of more than 0.005% across all samples. Any genus and species less than 1% that not mentioned here can be found in Supplementary files S2 and S3.

Figure 2 showing the overall taxonomy of microorganisms present in honey from phyla to species from each location. The fungal Amplicon Sequence Variants (ASVs) were aggregated from phyla to species exhibiting an abundance exceeding 0.005% were selected for inclusion in the honey composition plot. Three prominent phyla, namely Ascomycota, Basidiomycota, and Mortierellomycota, were identified across all honey samples together with their class, order, and family for each honey sample. However, their composition and abundance exhibit variations in each specific honey sample. Notably, the fungal phylum Ascomycota was the sole taxonomic group shared among all samples from Lombok, Bali, and Banggi, whereas Basidiomycota were dominant in Bali and Bangi honey only (Supplementary file S1).

At phylum level, all replicates of Lombok honey samples (B1) were dominated by the phylum Ascomycota (99.61%), followed by Basidiomycota (0.34%), while Mortierellomycota accounted for 0.04%. At the genus level, the Lombok honey samples were dominated by the genera *Zygosaccharomyces*, *Aspergillus*, and *Aureobasidium*, with relative read abundances of 68.81%, 29.76%, and 0.46%, respectively. At the species level, the predominant species was *Zygosaccharomyces mellis*, representing 68.55% of the total read abundance. Subsequently, *Aspergillus penicillioides* was identified as the second most dominant species with a relative read abundance of 27.66%, while *Aspergillus arenarioides* represented the third dominant species with a relative read abundance of only 1.19% of the total reads (Fig. 2). Similarly, the mycobiota of Bali (B2) honey primarily comprises Ascomycota (71.46%), followed by Basidiomycota (28.18%), with Mortierellomycota accounting for a minor fraction (0.37%). Analysis at the genus level reveals the presence of nine dominant genera in Bali (B2) honey samples. Genus *Aureobasidium* dominates with a relative read abundance of 40.81%, other genera like *Aspergillus*, *Cystobasidium, Exophilia, Cerrana*, *Rhodotorula, Trichoderma*, *Penicillium, Phlebiopsis*, *Peniophora, Magnibotryascoma*, *Lulwoana, Tinctoporellus, Epithele,* and *Xylodon* represent 1–5% of the relative read abundance. Further examination at the species level indicates that *Aureobasidium pullulans* is the predominant species in Bali (B2) samples, constituting 40.1% of the relative read abundance, other species like *Cystobasidium calyptogenae, Exophiala oligosperma, Cerrana unicolor*, *Peniophora crassitunicata*, *Rhodotorula mucilaginosa, Peniophora malaiensis, Tinctoporellus bubalinus*, *Magnibotryascoma kunmingense*, contribute minimally, each representing 1–5% of the relative read abundance (Fig. 2) (Supplementary file S3).

In comparison, the phylum-level analysis of Bali honey samples (B3) reveals a different composition, with Basidiomycota accounting for the majority 61.87%, followed by Ascomycota (37.7%), and Mortierellomycota (0.53%). Dominant genera Bali honey (B3) is represented by *Aureobasidium (*25.13%), second highest genera *Cerrana (*8.77%), other genera *Zygosaccharomyces, Aspergillus*, *Cystobasidium, Exophilia, Rhodotorula, Phlebia, Trichoderma*, *Penicillium, Trametes, Tinctoporellus, Schizophyllum, Letendraea, Montagnula, Physisporinus, Hortaea, Trichomerium, Talaromyces*, and *Fomitopsis* represent 1–5% of the relative read abundance. At level species, *Aureobasidium pullulans* remains the dominant species in Bali (B3) honey, constituting 25.13% of the relative read abundance, followed by *Cerrana unicolor* (8.77%). Notable contributions from other species, including *Zygosaccharomyces rouxii, Cystobasidium calyptogenae, Exophiala oligosperma, Rhodotorula mucilaginosa, Penicillium echinulonalgiovense, Trametes cubensis, Tinctoporellus bubalinus, Schizophyllum commune, Letendraea cordylinicola, Hortaea werneckii, Trichomerium lapideum, Trametes elegans,* and *Gliomastix murorum*, represent 1–5% of the relative read abundance (Fig. 2).

Comparative analysis between Bali (B2) and Bali (B3) honey samples reveals both overlapping and distinct characteristics in their mycobiota. While both samples share *Aureobasidium pullulans* as the dominant species, with varying relative abundances of 40.1% in (B2) and 25% in (B3) respectively, while differences in phylum-level dominance are evident, with Ascomycota being predominant in B2 and Basidiomycota in B3. Additionally, variations in the relative abundances of several genera and species are observed between B2 and B3. For instance, *Rhodotorula* is significantly more abundant in B3 (8.7%) compared to B2 (1%). These findings suggest that while both samples share microbial components, specific factors may influence their mycobiota composition. Factors such as floral sources, collection time, and storage conditions may contribute to the observed variations in microbial communities. Thus, despite their geographical proximity, B2 and B3 honey samples exhibit distinct mycobiota compositions, reflecting potential influencing factors unique to each sample.

In Banggi honey (B4) sample, the phyla Ascomycota dominates at reads abundance 90.09%, followed by Basidiomycota at 9.79% and Mortierellomycota with only 0.11% of relative read abundance. At genus level the Banggi honey over dominant by *Zygosaccharomyces* (45.17%), followed by 2nd highest genera *Aureobasidium (*35.24%). Other genera like *Aspergillus, Cystobasidium*, and *Exophilia* represent 1–2% of the relative read abundance. The predominant species in Banggi (B4), honey samples are highly dominant by *Zygosaccharomyces rouxii* (42.71%), followed by 2nd highest dominant species are *Aureobasidium pullulans (*35.49%). Other genus like *Cystobasidium calyptogenae, Exophiala oligosperma,* represent only 1–2% of absolute read abundance (Fig. 2).

The examination of honey samples obtained from Lombok, Bali, and Banggi demonstrates clearly differentiated fungal compositions. Although Ascomycota is found all over, Basidiomycota is only dominant in Bali and Banggi. The predominant species found in Lombok honey is *Zygosaccharomyces mellis*, along with the presence of *Aspergillus penicillioides.* These microorganisms, *Zygosaccharomyces* and *Aspergillus penicillioides*, are distinctive features of Lombok honey. *Aureobasidium pullulans* is the predominant microorganism found in Bali honey, while *Zygosaccharomyces rouxii* is the dominant microorganism in Banggi honey. These findings emphasize the impact of geographical factors on the variety of fungi found in honey, emphasizing the significance of comprehending changes in microbiota for purposes of quality control and health evaluation.

## Methods

### Collection of honey samples

**Figure 3:**
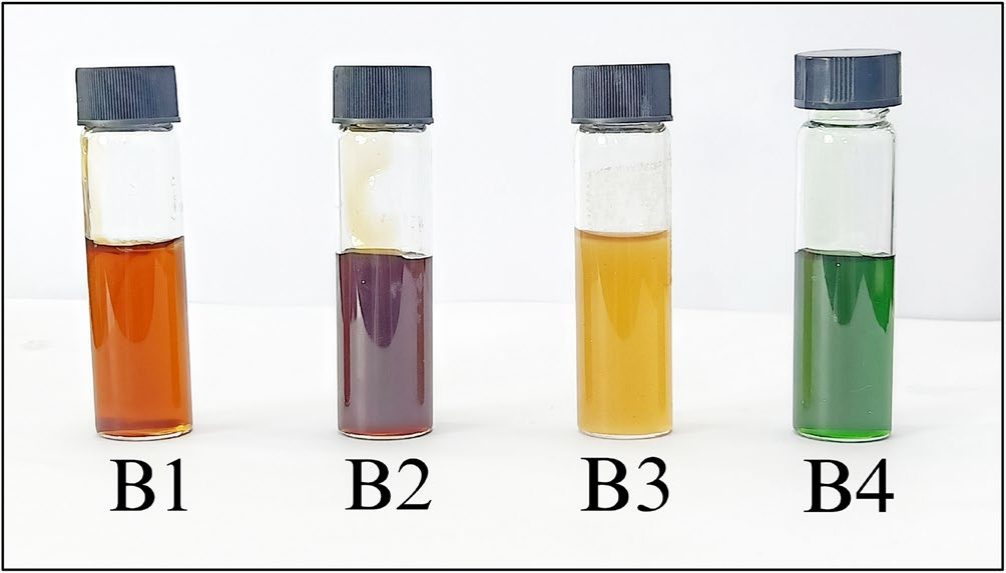
The honey samples collected from distinct geographical locations: Lombok (B1 yellow gold), Bali (B2 dark gold, and B3 light gold), and Banggi Islands, Sabah (B4 dark green).

The four honey samples were obtained from each origin (Table 3) harvested in the months of early August 2023 to mid of November 2023. The honey samples were collected in biological quadruplicates from each location. The freshly collected honey was labeled as follows: Lombok (B1), Bali (B2 & B3), and Banggi Island (B4) (Fig. 3). All honey samples were stored in screw-capped dark containers at room temperature (approximately 25 °C) prior to analysis.

**Table 3:** Details of honey samples utilized in the study.

Honey	Province	Coordinate
B1	Madu Lombok Utara, Desa Sukadana, North of Lombok	(8° 13′ 16.824″ N, 116° 23′ 58.200″ E)
B2	Raw Bali honey was obtained from the local private Honeybee farm at Desa Kuwum, Badung Regency, Bali	(8° 13′ 16.824″ N, 116° 23′ 58.200″ E )
B3	Tenganan, Manggis, Karangasem Regency, Bali	(° 29′ 07.3″ S 115°33′ 45.5″ E)
B4	Banggi Island, Sabah	(7° 12′ 43.1028″ N, 117° 7′ 18.9948″ E )

### eDNA preparation from honey

The extraction of DNA was performed with little modification as described previously^2,3^. A 50 g sample of honey was divided into four 50 mL Falcon tubes, each containing 12.5 g of honey. Subsequently, 2 mL of ultrapure water was added to each tube. The solutions were then incubated at a temperature of 65 °C for 30 min while being stirred. Following centrifugation at a force of 12,000 × g for 20 min, the liquid portions located above the mixture were removed and discarded. The pellet samples were resuspended and mixed in a solution consisting of 1 mL of ultrapure water and 1 mL of phosphate-buffered saline (PBS). The resulting mixture was then transferred to a 2 mL centrifuge tube and subjected to centrifuged at 12,000 × g for another 20 min. The liquid portion was disposed of, while the solid residue was preserved at a temperature of − 20 °C. For further DNA extraction experiments, the Zymobiomics DNA Extraction Kit (Zymo Research, CA, USA) was utilized following the manufacturer’s instructions.

### Library preparation and sequencing

The fungal ITS1 region was amplified using the primers BITS (5′ACCTGCGGARGGATCA3′) and B58S3 (5′GAGATCCRTTGYTRAAAGTT3′)^21,22^. An additional 5 bases of inline barcode were incorporated at the 5’ end of the primers to enable inline barcoding. Different samples were amplified using different combinations of the forward and reverse inline primers. PCR was performed using SolarBio 2X Taq PCR MasterMix (SolarBio Life Sciences, Beijing, China) with the PCR profile of 95 °C for 3 min followed by 35 cycles of 95 °C for 15 s, 48 °C for 20 s and 72 °C for 10 s with final extension at 72 °C for 5 min. The barcoded amplicons were subsequently visualized on an electrophoresis gel. In each experiment, the PCR amplification is deemed successful only when samples exhibit a discernible band of the correct size on the gel, and the negative control displays neither the anticipated band nor primer dimer and subsequently purified using 0.8 X of solid-phase reversible immobilization (SPRI) bead. The purified amplicons were used as the template for 8 cycles of index PCR to incorporate the complete Illumina adapter and Illumina-compatible dual-index barcodes. The constructed libraries were subsequently size selected using 0.8 X vol of SPRI bead and pooled into a single tube. Quantification of the pooled libraries used Denovix DsDNA High Sensitivity Assay (DeNovix Inc, DE, USA). Sequencing of the pooled libraries was performed on a NovaSEQ6000 (Illumina, San Diego, USA) using the 2 × 150 bp paired-end sequencing configuration.

### Data analysis

Demultiplexing and primer trimming of the raw paired-end reads used cutadapt v1.18^23^. The trimmed reads were subsequently merged using fastp v0.21^24^. The processed reads were imported into QIIME2 v.2022.8^25^ for further analysis. Amplicon Sequence Variants (ASVs) were obtained using the dada2 v1.22 R package^26^. Subsequently, potential contaminants were identified in silico from the dataset using the R package DECONTAM^27^. Despite applying a conservative threshold value of *p* < 0.10 to identify potential contaminants, the dataset under consideration met this criterion, and consequently, no taxa were excluded from the dataset. Taxonomic assignment of the ASVs was carried out using q2-feature-classifier by Bokulich et al.^28^, which has been trained on the latest UNITE database (unite_ver9_dynamic)^29^. Only ASVs with taxonomic assignment at least to the phylum level were selected for subsequent analysis. The ASV table and taxonomic classification table were exported using QIIME2 tools into tab-separated values (TSV format) and manually formatted to generate Microbiome Analyst-compatible input^30^. This prepared data was utilized for various analyses, including SparCC co-occurrence network construction and statistical analysis employing the linear discriminant analysis (LDA) effect size (LEfSe) method^31^. Alpha- and beta-diversity was calculated using specialized QIIME2 plug-ins. To gain insights into the relative abundances among taxonomic hierarchies, a filtered relative abundance table was used as the input for analysis^32^.

## Discussion

The foraging behavior of honeybees exposes them to a diverse array of habitats, facilitating encounters with various environmental DNA sources. This interaction gives rise to an intricate environmental DNA (eDNA) signature that permeates the honey they produce. Honey serves as more than just a medium for external DNA; it also reflects the ecological dynamics within the superorganism of the hive and its internal conditions. Throughout the process of honey production, from its initial development through maturation to final production, DNA is acquired from all biological components involved^2,13^. The microbiome of honey encapsulates valuable bioindicators, shedding light on factors such as the agricultural and urban landscape, the microbial milieu honeybees are exposed to, and even the chemical contaminants encountered during foraging journeys^8^. Furthermore, the botanical origin of honey significantly influences the composition of its fungal community^1,3^. Metabarcoding techniques, such as ITS1 sequencing and other next-generation sequencing methods, play a crucial role in unraveling the intricate web of mutualism and symbiosis woven by honeybees within their ecosystem^8,13^. The utilization of ITS1 metabarcoding, coupled with Illumina sequencing, enables a comprehensive taxonomic classification of the honey mycobiome. This not only serves as an indicator of overall beehive health but also offers insights into the origin and authenticity of honey^13^.

In essence, the integration of molecular techniques into honey analysis not only enhances our understanding of honeybee ecology but also holds significant potential for ensuring the quality and authenticity of this natural product. Using alpha diversity metrics such as Chao1, Shannon index, and Simpson index, we were able to assess both the richness (number of taxa) and evenness (relative abundances of those taxa) within individual honey samples. In contrast, beta diversity indices like RPCA, Bray Curtis, Jaccard weighted, and unweighted allowed us to explore the variations and taxa identified among different honey samples^33^. Among the honey samples analyzed, those from Bali (B2) exhibited the highest diversity, as determined by the Chao1 index. Bali honey (B3) followed as the second most diverse, consistent with previous findings indicating that honey composition is influenced by its origin^3^. While both types of honey shared certain taxa, they differed notably from Banggi and Bali honey in fungal composition. Shannon entropy analysis revealed that Bali (B3) displayed higher alpha diversity in terms of both richness and evenness. Simpson diversity indices highlighted differences in the dominance or evenness of microbial species within the honey samples. Lombok honey exhibited a higher Simpson index, indicating a more dominant presence of certain microbial taxa, likely influenced by specific environmental factors or unique floral sources in the region. This analysis represents the first quantification of fungal beta diversity among different habitats across neighboring countries such as Sabah (Malaysia), Bali and Lombok (Indonesia).

The highest dissimilarity in fungal beta diversity was observed using the RPCA metric (91.56%), followed by the Bray Curtis metric (71.98%) among honey samples. These results emphasize the significant differences in fungal composition among honey samples, highlighting the potential for identifying honey origin^3,13^. Lombok honey (B1) occupied the left side of the Bray Curtis plot, while Banggi honey was positioned on the right side, indicating their distinctiveness from each other. In contrast, (B3) and (B4) were situated closer to each other or in the middle of the plot, suggesting similarities in mycobiota and origin. These beta diversity results indicate that honey samples from various origins not only differ but also exhibit varying species abundances within unique fungal communities. This is likely influenced by multiple variables such as climate, floral sources for pollen gathering, and beekeeping practices.

The phylum Ascomycota is the sole dominant group across all honey samples (see supplementary file S1), indicating a highly diverse fungal community at the genus level. Comparing honey samples at the genus level, several genera such as *Aureobasidium, Aspergillus, Cystobasidium, Exophiala, Cerrena, Rhodotorula, Eurotiales, Phlebia, Trichoderma, Penicillium, Phlebiopsis, Lulwoana*, and *Trametes* show high resemblance. Additionally, unique genera such as *Peniophora, Epithele, Vishniacozyma, Cladophialophora, Magnibotryascoma, Steccherinum, Coriolopsis, Epicoccum, Scopuloides, Nigrograna, Corynespora,* and *Neooccultibambusa* are found only in Bali honey samples (B2 and B3), distinguishing them from other samples (see supplementary file S2). At the genus level, *Zygosaccharomyces* is highly abundant only in Lombok and Green honey samples, indicating a healthy yeast growth environment. Previous studies have also isolated *Zygosaccharomyces* from honey using both culture-dependent and non-dependent methods^34^.

*Aureobasidium* a dominant genus in Bali (B2 and B3) and Banggi honey (B4), likely due to its presence in the phyllosphere and carposphere of various fruits and vegetables, which honeybees easily transfer. This is consistent with previous findings^9,35^. Notably, *Aureobasidium* is identified for the first time in Banggi honey (B4) sample. A xerophilic fungus, is highly abundant in Lombok honey (B1) compared to other samples, as it thrives in environments with low water activity^36^ (see supplementary file S3). *Zygosaccharomyces rouxii* and *Zygosaccharomyces mellis* are dominant species within the Zygosaccharomyces genus, with *Zygosaccharomyces mellis* being highly abundant in Lombok honey (B1) and *Zygosaccharomyces rouxii* in Banggi honey (B4). The diversity observed in fungal communities across honey samples highlights their varied origins and hosts, corroborated by existing literature^1,3,13^.

The presence of *Zygosaccharomyces, Aureobasidium*, and *Aspergillus* in honey samples is not only common in the current study but also widely reported in other literature^1,8,9^. This prevalence can be attributed to their ability to tolerate the unique environmental conditions of honey. These organisms possess traits such as osmotolerance, xerotolerance, and acidotolerance, which allow them to thrive in environments characterized by high sugar content, low water activity, and low pH, respectively. Consequently, they are well-suited for survival and proliferation in honey, which typically exhibits these properties. *Zygosaccharomyces, Aureobasidium*, and *Aspergillus* are known to inhabit various natural environments, including soil, plants, and decaying organic matter, suggesting their adaptability to diverse ecological niches, including the floral sources of honey. It is believed that these organisms may enter honey through nectar sources. During the collection of nectar from flowers, bees may inadvertently transport fungal spores present in the environment, which subsequently contaminate honey during the production process. *Aureobasidium* and *Aspergillus* are sometimes associated with honey contamination. External sources, such as the bee foraging environment, can introduce fungal spores into honey from decaying plant matter, leading to contamination during harvesting and processing. The use of common metagenomic analysis worldwide has facilitated the application of culture-independent methods, such as metagenomic analysis, in studying the microbial composition of honey. This approach has revealed the presence of these fungi in honey samples, even at low abundance, thereby offering a more comprehensive understanding of the microbial community present in honey. Despite the valuable insights, certain limitations must be acknowledged. The sample size was limited due to the restricted number of reliable honey producers in Bali, Lombok, and Banggi Island, and the unique green honey (B4) from Sabah is found only on Banggi Island. These constraints limited the number of samples that could be collected and analyzed. Additionally, the collection timeframe, from early August 2023 to mid-November 2023, represents a specific period within the honey production season. Consequently, the findings provide a snapshot rather than a comprehensive analysis of the temporal dynamics of the honey microbiome.

## Conclusion and future perspective

Current study demonstrates the effectiveness of ITS1 analysis in accurately distinguishing honey samples from different regions, highlighting its potential as a valuable tool for honey authentication. The findings affirm the superiority of DNA-based identification methods over traditional techniques. Notably, honey samples from Bali exhibit significantly higher levels of alpha and beta diversity compared to samples from other regions, highlighting a rich and unique microbial composition. These results emphasize the importance of geographical variation in honey quality and the need for precise authentication methods in the honey industry. The distinct microbial profile of Bali honey suggests unique environmental and floral influences. Recognizing the limitations of the current work future research should aim to include a larger and more diverse set of honey samples collected over extended periods and under varying environmental conditions. This approach will provide a more comprehensive understanding of the dynamic changes in the honey microbiome. Additionally, integrating physical and chemical analyses of honey samples will offer deeper insights into the factors influencing microbial diversity. These efforts will enhance in understanding of honey microbiomes and their implications for product quality and authenticity in the honey industry.

### Supplementary Information

Supplementary Information 1.

Supplementary Information 2.

Supplementary Information 3.

## Data Availability

Sequence data for this study were uploaded to the NCBI SRA database under Project (PRJNA1036045) with the following accession number: (a) SRX22424189 Lombok Honey (B1): Fungal species identified in honey samples from Lombok Island, Indonesia. (b) SRX22424192 Bali Honey (B2): Fungal species identified in honey samples from Bali Island, Indonesia. (c) SRX22424192 Bali Honey (B3): Fungal species identified in honey samples from Bali Island, Indonesia. (d) SRX22424191 Banggi Honey (B4): Fungal Species identified in Honey samples from Banggi Island, Sabah.
